# Progress in State Medicine

**Published:** 1902-02-15

**Authors:** 


### PROGRESS IN STATE MEDICINE.

### CANCER.

NEWSHOLME,[1] writing on the statistics of cancer mentions that in the five years, 1891-5, this disease was responsible for an annual registered mortality of 712 and in 1896 of 764 to every million of the population. It is almost certain that these figures understate the actual death-rate from malignant disease. Cancer causes more deaths every year than any other group of disease, with the exception of bronchitis, pneumonia, and phthisis. According to the figures for 1896 one out of every 14 men and one out of every nine women reaching the age of 35 years eventually dies of cancer. He draws attention to Payne's explanation of this increase, which is that the greatly diminished mortality from phthisis and from infectious diseases, both acting much earlier in life than cancer—a larger number will survive so as to be the victims of cancer at a later period of life. In 1881-90 the average death-rate from cancer of males aged 25 years and upwards was 9·49 per 1,000; in 1871-80 it was 6·97 per 1,000 of the population aged 25 and upwards. Considering the effect of occupation on cancer mortality he quotes Tatham's report, which for the period 1881-90 shows that among all occupied males the comparative mortality figure for cancer was 44, among all unoccupied males 96. The theory that excessive nervous strain and anxious work provoke cancer does not receive support in the fact that the comparative-mortality figure for medical men is only 43, while it is as high as 70 for brewers. The contrast between lawyers, 60, and clergymen, 35, is great. Chimney-sweepers occupy a supremely high position in mortality from cancer. The low figure for coal-miners indicates that they enjoy a comparative immunity from cancer, as well as from phthisis. He suggests that the high figures against some occupations may be attributed to intemperate habits. The greater increase of cancer mortality among males confirms the view that the registered increase of cancer mortality is in a large measure only apparent. Between 1881 and 1891 the registered increase of mortality from cancer was 69 per cent. among males, and 28 per cent. among females. This is explained by more accurate diagnosis, more frequent autopsies, and the fact that cancer in the male is more frequently internal and difficult of diagnosis than in the female. The question whether cancer is really on the increase could be solved were all deaths accurately certified, and especially if the primary site of the cancer were stated in each case. Park[2] states that cancer is increasing, not only in New York State, but all over the world, and is likely to do so, until its minute causes are positively determined. In England and Wales the cancer death-rate has risen from 1 out of 5,646 of population in 1840, to 1 out of every 1,306 of population in 1896. In New York State in 1887 there were 2,363 deaths from cancer, and 11,609 from consumption; in 1898 there were 4,456 deaths from cancer, and only 12,552 from consumption. He is not of opinion that this increase is due to improved methods of diagnosis. If for the next ten years the relative death-rates are maintained, we shall find that ten years hence, viz. in 1909, there will be more deaths in New York State from cancer than from consumption, small-pox, and typhoid fever combined. Referring to the cause of cancer, he believes that, considering its occurrence in certain families, and its prevalence in certain localities, it permits of scarcely any other explanation except the transmission from one person to another of a "contagium vivum" of some kind. The experiments to prove this have for the most part been carried on in a most traditional and conventional way, which renders them worthless. Inoculation work must be carried out along three lines of experimentation :—(1) From animal to animal ; (2) from the dead subject to the living animal ; (3) inoculation into animals of pure cultures of organisms taken from fresh human specimens. He is convinced that cancer is actually contagious. Haviland,[3] discussing the medical geography of cancer, draws attention to the fact that nearly the whole of Wales and the north-west portion of England show a low mortality. These parts belong geologically to the oldest formations, namely, the silurian and the carboniferous, and physically include the highest and best-drained mountainous districts in the country. The next great low-mortality group is to be traced from the south to the north-east ; it extends northward from the Isle of Wight, through the greater portion of Hampshire, Wiltshire, northwards to the Cotswold Hills, eastward to pass through Oxfordshire, Bedfordshire, Hertfordshire, Cambridgeshire, and Norfolk. These two great groups and their intermediate links are the elevated districts of England, and they contain the sources of the great rivers which drain the country. On the other hand the high-mortality districts are found to surround the great rivers after their full formation, and when they have reached the low-lying valley lands, where the districts through which they pass are liable, after heavy rains or sudden thaws, to serious floods. In East Yorkshire, the Midland Counties, and along the course of the Thames, floods constantly occur, and these areas are all coincident with the districts of high cancer mortality. Power,[4] having investigated a large number of cases with special reference to local distribution of cancer and cancer houses, says that these cases raise a strong presumption in favour of cancer being an infective disease, to which some are more susceptible than others. Locality seems to be a predominant factor, and the nature of the locality associated with cancer appears to be so characteristic that it is possible to say whether or not it is advisable for a patient with a well-marked family history of malignant disease to reside in a given spot. He does not think that the cause will be found in any given room, house, or water-supply ; rather that it will be proved that there is some intermediate host, whose chance of detection will increase or diminish with the care which is taken to examine the fauna and flora of the districts where cancer is most prevalent. Major,[5] whilst strongly of opinion that there has been a real increase of cancer, is not prepared to accept the cancer-mortality statistics on account of defective death certification. He quotes the fact that during the year 1896 letters of inquiry were sent by the Registrar-General to practitioners with respect to unsatisfactory certificates, which inquiries resulted in as many as 597 deaths being transferred from various vague causes to the cancer list in one year. In 1889, 421 cases were transferred similarly ; and in 1896, out of 111 deaths returned as due to stricture of the œsophagus, 74 were upon investigation considered to be cases of cancerous disease, and corrected accordingly. Earnest endeavours should be made to verify diagnosis by post-mortem examination. The report[6] of the Cancer Committee of the Birmingham branch of the British Medical Association deals mainly with this disease in Warwickshire, Staffordshire, Shropshire, and Worcestershire. Medical men in the different registration areas of these counties were asked to make lists from the official registers of all deaths certified as due to malignant disease during the previous ten years, and in the second place to give details of the localities both where the disease was more prevalent and also where less so, having particular regard to the dryness and sewage contamination of the soil, and the general condition of those houses where malignant disease had occurred with marked frequency. Returns have been utilised from 50 registration areas, representing a population of 860,000 persons, and including more than 5,300 cases of malignant disease. The report states that there are certain more or less well defined areas in which the mortality is markedly higher than the average, and others in which it is much lower—a damp, ill-drained, water-logged soil is more frequently associated with a high cancer death-rate than a dry well-drained soil—and that contamination of the soil or subsoil for longs periods with decomposing organic matter is a concomitant of, and probably bears a causal relation with, the specially high cancer mortality ; that while second and third cases of cancer occur in particular houses with a somewhat greater frequency than can be accounted for by mere coincidence, there is much greater weight of evidence that cancer is found with extraordinary frequency in particular groups of houses, say, in the same street, thus constituting "cancer foci." This may be associated with contaminated soil. Cancer occurs more frequently in old than in new houses, and in districts inhabited for a long time than in those recently built over. It is suggested, from evidence collected, that under certain circumstances the disease may be transmitted direct from one person to another among those who are constantly in close association. Behla[7] made some researches on the merulius lacrymans, the cause of "dry rot," because he had noticed that the houses in which cancer was most common were those with dark and damp cellars, in which "dry rot" was abundant. He was unable to demonstrate any pathogenic property in this organism.

[1] Practitioner, April, 1899. [2] Ibid. [3] Ibid. [4] Ibid. [5] Brit. Med. Jour., July 20, 1901. [6] Brit. Med. Jour., Sept. 29, 1900. [7] Brit. Med. Jour., May 25, 1901.

